# The Mutual Relations of the Medical Profession, Its Press, and the Community

**Published:** 1871-06

**Authors:** Horatio Robinson Storer

**Affiliations:** President of the Association


### THE MUTUAL RELATIONS OF THE MEDICAL PROFESSION, ITS PRESS, AND THE COMMUNITY.

AN ADDRESS DELIVERED AT THE ANNUAL MEETING OF THE ASSOCIATION OF MEDICAL EDITORS, ON MAY 1ST, 1871, AT SAN FRANCISCO.

By HORATIO ROBINSON STORER, M.D., President of the Association.

*Gentlemen of the Association of American Medical Editors:* Coming together from the opposite portions of the Continent, we have met to-night, not merely "to cultivate professional courtesies, and to facilitate the conduct and general management of our journals," but, still further to quote the language of our constitution, "to promote their usefulness, and make them a still greater power for professional and popular good, and thereby, most especially, "to advance the interests of Medicine." Such being the purpose and intent of our organization, there can be no topic more appropriate for me to present to you; none more fitting to the time, the place, and all the circumstances of the occasion, than

THE MUTUAL RELATIONS OF THE MEDICAL PROFESSION, ITS PRESS, AND THE COMMUNITY.

These relations are manifold. To consider them all would be impossible in the brief space of an half hour's address. I shall, therefore, endeavor to speak only of the most important of them, and, avoiding all attempt at fine writing, to make my remarks terse, very plain, and thereby, I trust, effective.

The Medical Profession in this country consists of what? To this question a multiplicity of answers present themselves, all of them true to a certain extent, and yet all of them, save one, very degrading to the term's highest idea. Were every physician what he should be—a thoroughly honest, straightforward man, anxious only for his patients' welfare, laboring for the development of his science, and not alone for gain, liberalized by education, humanized in the highest sense by a constant entering into the sufferings he is compelled to meet, and, above and beyond all else, spiritualized by the recognition that his every success is but a vouchsafement of God's great mercy, and he but its humble instrument—what a different art were medicine, what a different place in the world.

Of the seventy thousand or more persons in the United States licensed under the Revenue laws to practice medicine, how large a proportion, is it supposed, can be claimed to possess the qualifications just adverted to? Even if we eliminate all who, in default of professional graduation, have no valid title to the name, and all professional empirics, of whatever stripe or hue—Caucasian, aboriginal, or Chinese; tackers, whether of "path" or "ist" to their names—there still remains a mighty host, swelled again to its original dimensions, if the title is permitted, as in many sections of the country, to dispensing druggists, and still again to that doubtful sex wearing the habiliments of womanhood, but assuming the work and the prerogatives, while it seeks to escape the legal responsibilities, of man. * * * *

As a graduate in law as well as in medicine, from the twin schools of that dear old University whose foundation goeth back to the time when jurisprudence and the art of healing, those best transplantations of civilization, first were landed on the Atlantic coast, I yet yield for them the palm to that nobler vocation, by whose teachings and ministrations, though God's grace, our yokes here are lightened, and hereafter our best hopes ensured. "Christo et Ecclesiæ." To these did John Harvard dedicate his worthy gift, whose ever-recurring power manifests itself in the skill, the intelligence, and the professional reputation of so large a proportion of American medical practitioners. Do I say that the lawyer and the physician should yield precedence to the priest? Can any one of us who has personally looked within the veil, losing wife or child, or himself sick nigh unto death, do otherwise?

By such recognition of the true sacerdotal function, confessing our dependence upon that One Supreme, to whose immediate presence with us we owe every so-called cure, we but increase, with our own happiness and self-respect, our esteem by others; for what sceptic even is there who would not sooner trust his life to a devout physician than to an unbeliever? Or what man or woman who can afford in their direst strait to spurn the gentle, loving touch of the All-healing hand? * *

The physician is to render to his patient of the tenderness and sympathy, care and assistance he has himself received. Let every man see to it that the Fountain is not forgotten, nor impute thereto his own defilement of the precious stream.

That the education of physicians is frequently so limited goes far, there can be no doubt, to prevent that general bestowal of confidence which otherwise would be conferred. For this, however, the community partly, as in part ourselves, are to blame. If a second-rate article is all that is sought by the purchaser, he should not complain if it be received. If the medical colleges are content to underbid each other, and year after year to pursue the suicidal warfare, they should not grieve that their students, become practitioners, so often are starvellings, and so frequently do them discredit. * * * * *

Professional "intuition" in the treatment of disease is seldom to be found. It is a very different thing from the *vocation* of which I have already spoken—without a sense of which none should ever assume so sacred a trust. A knowledge of human nature is useful to us, as a matter of course. It no more, however, constitutes a complete preparation for practice than would a knowledge of mechanics, or of inorganic analysis. It is as with houses built upon a rock and upon the sand—unless early education be well laid and solid, a broad and good foundation, the most elaborate after-structure will prove easily shaken and unsafe. It matters not what, or how many, the apparent exceptions to this rule, for these brilliant self-made men would have shone with far more lustre had they but received the early training of whose lack none are more painfully conscious than themselves. However great is the credit their due, there's always a blur to the gem, and sometimes the very contrast with what might have been, makes this seem the greater. President Eliot, of Harvard University, told but the truth in that now famous paper of his, upon "The New Education." "The term, 'learned profession,'" he said, "is getting to have a sarcastic flavor. Only a very small proportion of lawyers, doctors, and ministers, the country over, are Bachelors of Arts. The degrees of LL.B. and M.D. stand, on the average, for decidedly less culture than the degree of A.B., and it is found quite possible to prepare young men of scanty education to be successful pulpit exhorters in a year or eighteen months. A really learned minister is almost as rare as a logical sermon." And as for the yearly graduates from our medical schools, "Poor humanity," continues President Eliot, "shudders at the spectacle of so large a crop of such doctors." Who of you will not admit that a really learned physician, in the highest sense, is as rare as, by differentiation, the only possible method, a perfectly correct abdominal diagnosis,—which, I am sometimes inclined to say, has never yet been made.

Such being the truth, what of ourselves—to a certain extent representative members of the profession—and of the power which we wield, the press? As individuals we may be very far from the standard our responsibilities demand—many of us undoubtedly are—but, in the aggregate, there's a mightiness in this editorial function, that makes of one's chair well-nigh the throne of Jove. Woe to the evil-doers upon whom its bolts chance to descend!

The opportunities and the influence of the Medical Press, its history in this country, and the causes which, thus far, have interfered with its full measure of usefulness, were all so intelligently discussed by my predecessor, Dr. N. S. Davis, of Chicago, that I will not weary you by their recapitulation.

A few words, however, may be necessary, in this connection, to render more evident the bearing of what will follow.

As there are many classes of so-called physicians, with but one real and honest distinctive type—so this expression "Medical Press" may mislead, unless now more strictly defined. Many of you are authors of no mean repute; you have published, out of the stores of your own experience, manuals or text-books in the several departments of medicine, or have laid your contributions, in the form of original memoirs or mongraphs, upon the lap of our science. Others, of whom the number was formerly far greater, have descended to a lower plane, and as translators or copyists, have revamped the work of foreigners into our English tongue—doing it too often, I grieve to say, as veritable pirates, without the slightest concert with the authors themselves—thus bringing the whole editorial profession into grave disrepute. * * * *

In our calling, as in all others, there are strong and positive tendencies,—on the one hand, upwards; on the other, towards deterioration. In the union that we now commemorate, just as there is strength for us all, so will it be found that the purer tendencies to which I have alluded will be intensified, the less worthy ones diminish or be destroyed.

License, for instance, you will not tolerate, even while ensuring a truer freedom. Every leaning towards irregularity in practice, or towards its excuse or encouragement, as one man you will rebuke. Praise of self will find itself merged in an utter forgetfulness of self-contemplation, through the very working for others' good.

In this connection I would say one word concerning the relations that we hold to these patrons, our brethren of the profession itself. We have our work to do for them, and we all of us endeavor to do it well. They encourage us by their contributions to our pages, by kind messages in the letters they write to us, and, to an ever-increasing extent, by the money enclosures therein contained. And yet, though personally I have had every reason to be grateful upon each of the scores I have named, I am sure that you will agree with me when I say that the medical profession as yet falls far short of its duty toward the press. * * There are honorable exceptions, it is true, to the remark that I have made. The magazine under my own direction has a subscriber who wrote that the "Gynæcological Journal" was the thirteenth medical periodical that regularly came to his table; and this was a hard-working, over-driven physician, in a sparsley settled country district, with no leisure for study, it would seem, than that afforded while in the saddle upon his daily beat; and yet I will venture to say that this gentleman, by this means, kept himself better informed, more completely at a level with the prominent men of the day, than thousands of city practitioners, with greater wealth, more leisure, infinitely more pretentions, and far less liberality towards the members of this Association. There is not a physician in this country, I dare affirm, who would not each year obtain his money back again, at compound interest, were he to subscribe for and read with the most ordinary appreciation, a copy of each of the journals that we represent. One-half of the sum that most men throw away at auction sales, for stale and musty editions of authors now far behind the age, expended in subscriptions for the medical journals of the day, would not only do much for the continued education of our friends in practice, and keep their minds alive to the improvements in methods of study and treatment constantly being made, but it would tend infinitely towards a greater appreciation of and respect for our own native medical writers, who, through the channels of communication you offer them, are becoming recognised, as never before, by the profession of foreign lands.

Let us turn now to the relations of our profession and its press to the community.

There are many persons who look upon their physician as simply their servant, to be paid his wages, and not always when due, at their beck by day and by night, and to be discharged when the whim takes them, as summarily as their horses' groom. There are practitioners, on the other hand,—would that there were more of them!—who, while they look to the public for means of support, yet believe that the skilled laborer, in such a calling, is in every way worthy of his hire; and so far from considering themselves as favored by those who call them to set a limb or ward off a convulsion, hold that it is they themselves who confer the boon, and that the arduous and often repulsive labors thus undergone for others' sake are not to be balanced by gold. These views conflict, the one with the other. Both are to a certain extent wrong; but I should dishonor my calling did I not hold, as I do most devoutly, irrespective of any *esprit de corps*, that our own view of the question is by far the more correct one. That it is not universally accepted by the community is not owing so much to a lack of grateful sensibility upon its part, as to a cheapening by physicians of each other, and of themselves. The moment a medical man descends to underbidding or decrying his neighbor, that moment he becomes to the commonest intelligence a mere market man, to be haggled with, brow-beaten, or taken advantage of himself. Were the provisions of the code of ethics of the American Medical Association generally accepted as they are by members of the profession, even though not as yet connected with that national body, but known and appreciated by the community, our present relations would be very materially changed. It would then be understood, that so far from being merely a system of checks and counter-checks for self-protection, and to preserve the privileges of a guild, the code exists for the safety of the public, to prevent quackery and its reckless tampering with the lives of men; to keep for the sacred art, so far as possible, its character of self-sacrifice; and to ensure, through the physician's own effort, his retaining the intrinsic nature of a gentleman—refined, and so fit to deal with exquisite mental and physical derangement,—honorable, and so to be trusted as the friend in the sorest need. We are not permitted to dispense secret medicines though to do so were a royal road to fortune, neither may we patent a medical invention or discovery, however meritorious in itself it may be. This negation is not for the purpose of defending ourselves from each other, but to protect the community from the chance of our yielding to those ordinary temptations that surround all classes of men, and to ensure to it the full measure of every stream of beneficence of whose source we may perchance obtain the key.

Were these facts but better appreciated, there would be less distrust of physicians, and of their measures for the relief of suffering, and less complaint by them of the ingratitude of their patients. * * * *

But, I may be asked, is it possible for us to withstand, to any appreciable extent, the flood of empiricism that is now everywhere threatening to beat down and cover all the old landmarks? Unless we have faith that it is possible, we are unworthy to be here in California at the present moment, surrounded, as we are upon every side, by monuments to success under what seemed insurmountable difficulties; to courage that saw, in this begun, the same already accomplished.

That there exist in all communities, representatives of every form of irregularity in practice, what our Canadian neighbors call medical "sects;" that the present extreme tendency to popularize, upon the part of our more prominent professional writers, may bring dignity and permanence of standing into jeopardy; that the running riot of men's and women's minds in their discussions of questions of social science, whether within or without special associations provided therefor, goes far to confuse anew many a matter already none to plain—these are certainly discouragements. But what of that? Were everything plain sailing, were there no dangers to avert, and no obstacles to overcome, of what possible purpose would be our Association? Of what use indeed, our journals at all?

We object, very properly, to certain definite and distinct violations of the Ethical Code; to "irregularities" so-called, and every looking thereto; but we yet permit an extremely wide range of action. We would not advise that every man should be his own physician; he, himself, is often the first to recognize that error; precisely as when, his own lawyer, he attempts to manage a case in Court. And yet how much preferable it would be, did technical skill and what is known as common sense, oftener make each other's acquaintance! We believe in erudition; and yet, is he not the best general practitioner, who is, after all the best nurse? Was there ever a coroner's inquest where it would not have been for the public interests, had the jury understood a little better the scientific evidence underlying the case? Was there ever a trial for malpractice where justice did not feel the lack of a clearer insight into medical measures or surgical methods, and the still more mysterious processes of nature? * *

To-morrow we are to meet our subscribers and contributors from all parts of the country. They have given us aid and encouragement; we, in return, can stay their hands in their every effort for the increased influence and honor of the great national medical body. Many and varied will be the measures that are to be, or may be, proposed. There is the fundamental and ever-recurring question of Medical Education. Shall it still remain in the custody of the college teachers, who have found it difficult to be perfectly disinterested in this matter—there are many of them among ourselves, but as editors they have risen to a higher level, and I can therefore speak thus freely—or is it to be settled by the outside profession, which has already wisely decided that it has the power? In the letters which I have received from every one of your number, you have urged me, almost without exception, to declare, as the decided voice of the Editorial Association, that the standard of medical education in this country *must be raised.* * * *

Will it, again, be an advantage or not for the Association to recommend the formation of a National Medical School, liable as such would always be, through political changes and favoritism, to pass into the hands of the common enemy.

The establishing of a National Medical Journal, which, overshadowing that of our learned brother Cox, of Georgetown College, should, like the organ of the British Medical Association, serve as the especial mouth-piece of the great annual professional conclave.

The formation of a Board of General Scrutineers, whose gauntlet would prove far more fatal than those of the present

Annual Committee of Arrangements and the Committee upon Ethics combined, to many presenting themselves as delegates.

The founding of a National Board of Censors, with branches in every State, whose examination should stamp, as worthy or not, the standing of every physician already holding a college diploma.

Whether or no there should be a National System of Quarantine.

The upholding the Code of Ethics, as binding upon societies of medical men as well as upon individuals, and branding with infamy attempts, like that recently made by the Councillors of the Massachusetts Medical Society, to set it at naught.

These are all of them topics of the highest professional moment. In their settlement you have an interest, now by your votes, and hereafter in the fertile fields for discussion they are to afford your pens. I have no question that your influence, then and now, will be cast as an unit upon the side of the right. We legislate not for ourselves, but for the future. * * * * * * *

The Doctor then alluded to a conflict which had arisen in the East as to the controlling power of the American Association, and then proceeded as follows:

There are two points of great interest to us as journalists to which I will here call your attention. Together they comprise a means of reaching the profession collectively, and of placing the labors of our fraternity within its reach, to an extent never before possible. For them both, we are indebted to the unselfish and tireless industry of Dr. J. M. Toner, of Washington, for so many years a prominent member of the American Medical Association. Dr. Toner has prepared, and corrected to the present moment, a list of 50,000 of the physicians now practicing in the United States. This he places at your disposal, for consultation or other use. He is also engaged in preparing a complete index of the contents of all the medical journals hitherto published in the country. The value of this work, when completed, will be incalculable. * * *

The following is the register of the Association. At the time I assumed its Presidency, there were, as I have said, thirteen journals enrolled, of which one, the "St. Louis Medical and Surgical Reporter," has since ceased to exist. There remained, therefore, the following twelve:—

Chicago Medical Examiner; Baltimore Medical Journal; Richmond and Louisville Medical Journal; Nashville Journal of Medicine; Galveston Medical Journal; New Orleans Journal of Medicine; Detroit Review of Medicine and Pharmacy; American Practitioner (Louisville); Cincinnati Lancet and Observer; Oregon Medical and Surgical Journal; American Journal of Obstetrics and Diseases of Women and Children (New York); Journal of the Gynæcological Society of Boston.

In addition to the above there have joined us during the past year no less than twenty-six journals more; to wit:

New York Medical Journal; New York Medical Gazette; New York Medical Record; Journal of Psychological Medicine (New York); National Medical Journal (Washington, D. C.); American Journal of Insanity; Buffalo Medical and Surgical Journal; Medical Times (Philadelphia); Chicago Medical Journal; Indiana Journal of Medicine (Indianapolis); Michigan University Medical Journal (Ann Arbor); St. Louis Medical Archives; St. Louis Medical and Surgical Journal; Cincinnati Medical Repertory; Leavenworth Medical Herald; North-Western Medical and Surgical Journal (St. Paul, Minn.); Pacific Medical and Surgical Journal (San Francisco); Boston Journal of Chemistry; Physician and Pharmaceutist (N. Y.); Photographic Review of Medicine and Surgery (Phila.); Georgia Medical Companion (Atlanta, Ga.); Medical and Surgical Repertory (Griffin, Ga.); Kansas City Medical Journal; Clinico Pathological Reporter (Jefferson, Texas); Canada Medical Journal (Montreal); Canada Lancet (Toronto).

Gentlemen, you had my hearty thanks for the honor you conferred, far beyond my every poor merit, when electing me to this most honorable post. I now repeat them, for the courtesy extended to me upon the present occasion. In your behalf, also, I would express the gratitude of the Association to our California brethren for their kind welcome and most liberal hospitality.

May we return to our homes from this land of enterprise, rapid growth, and largeness of heart, educated, even by so short a sojourn, to a greater breadth of view, a more self-sacrificing zeal, and higher purposes, than a single one of us has ever known before. Our union will then have been cemented strongly enough to resist any and every force of demoralization, whether from without or within; and the profession, recognizing at last the power of our fraternity, will frankly confess, as has so long been done by the community at large, that the Press, well organized and wisely conducted, in reality rules the world.

